# Game-Theoretic Optimized Federated Learning for Heterogeneous IoT Object Detection

**DOI:** 10.3390/s26144420

**Published:** 2026-07-12

**Authors:** Zepeng Wang, Jie Chen

**Affiliations:** 1 School of Cyberspace Security, Gansu University of Political Science and Law, Lanzhou 730070, China; 2 Department of Automation, University of Science and Technology of China, Hefei 230027, China

**Keywords:** federated learning, game theory, heterogeneous IoT, object detection, complex networks

## Abstract

Federated object detection allows distributed IoT cameras to learn a shared detector without exposing raw images. Its performance is limited by non-IID scenes, unequal device resources, intermittent links, and selfish participation. We propose GO-FedDet, a game-theoretic optimized federated detection framework for heterogeneous IoT application. Client participation is formulated as a Stackelberg game, selection is formulated as an exact-potential resource game, and the equilibrium is embedded into a proximal detection objective. A utility-aligned aggregation balances detection contribution, communication/energy cost, and long-term fairness. We prove the existence of a Stackelberg equilibrium and the finite-improvement convergence of the selection game, and we derive a non-convex convergence bound under client drift and partial participation. Experiments on heterogeneous edge object detection show that GO-FedDet improves accuracy, lowers communication cost, and stabilizes fairness compared with representative federated baselines.

## 1. Introduction

Object detection over IoT vision networks underpins applications such as traffic perception, industrial inspection, smart agriculture, and edge surveillance [1,2,3]. Cameras and embedded sensors collect local scenes, but raw images are often private, bandwidth-intensive, and subject to site-specific policies [4]. Federated learning (FL) offers a natural paradigm where each client trains locally and a server aggregates model updates [5,6]. However, object detection is harder than image classification in FL: it couples localization and classification losses, relies on dense anchors or queries, and is highly sensitive to rare objects and background shift [7,8].

Recent studies identify FL as a core mechanism for privacy-preserving edge learning but also emphasize statistical heterogeneity, device heterogeneity, communication cost, and reliability as persistent obstacles [9,10,11]. Specialized studies have evaluated federated detectors for autonomous driving, UAV monitoring, maritime vision, and agricultural scenes [12]. These studies confirm the feasibility of collaborative detection, yet they usually treat client participation as a sampling problem rather than as a strategic interaction among resource-limited IoT nodes.

Client selection, incentives, and aggregation have been extensively studied [13,14,15]. Hierarchical aggregation, asynchronous coordination, interior-point client selection, and energy-aware FL improve scalability for heterogeneous networks [16,17]. Game-theoretic and blockchain-assisted incentive mechanisms indicate that rational clients may reduce participation without proper rewards, reputation, or contracts [18]. However, most approaches optimize a scalar training objective and rarely couple game-theoretic participation with the structured loss of object detection, and they also do not provide a unified convergence analysis for detection-oriented FL under selfish behavior [19].

Game-theoretic and resource-aware studies further show that FL over the IoT is shaped by incentives, congestion, energy budgets, and hierarchical edge coordination [20,21]. Voting-based and clustered selection mechanisms enable scalable coordination for massive networks of devices; federated few-shot detection, privacy-preserving object detection, and secure visual processing have emerged as recent research directions of interest [22]. Blockchain-assisted aggregation and reputation frameworks enhance reliability and trust [23]. From an optimization perspective, personalized FL, model-contrastive learning, matched averaging, and flat-minima regularization provide tools for handling statistical heterogeneity [24]. Classical game theory and potential games supply the equilibrium and convergence foundations used in this work.

The key novelty of this is not the independent use of a Stackelberg game, a potential game, or proximal FL but the closed-loop coupling of these components for federated object detection. Rather than select clients solely by data size or loss, GO-FedDet links four decisions that are usually handled separately: the server reward, the client’s willingness to participate, the congestion-aware channel-selection equilibrium, and the detection update used for aggregation. The Stackelberg game determines participation thresholds, the potential game converts feasible participants into a Nash-stable selected subgraph, and the resulting selection bias appears explicitly in the non-convex convergence bound. The proximal detection objective then limits local object-detection drift, while the fairness queue feeds long-term participation history back into both selection and aggregation [25,26,27]. This coupling provides a methodological contribution beyond a direct combination of existing modules.

**Figure 1:**
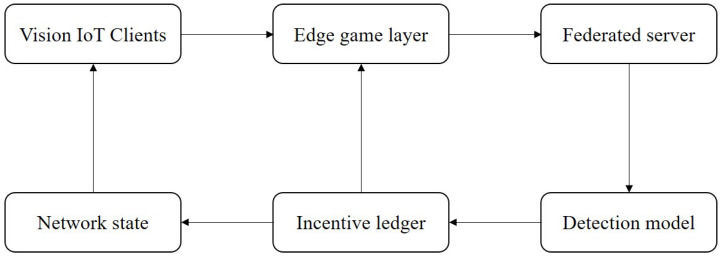
GO-FedDet framework for heterogeneous IoT detection.

This paper proposes GO-FedDet, a game-theoretic optimized federated learning framework for heterogeneous IoT object detection. In each round, the server posts a reward vector and a resource budget. Clients evaluate their marginal detection contribution, energy cost, communication delay, and historical participation. Selected clients train a detector locally with a proximal loss, and the server aggregates updates via a utility-aligned rule that combines validation gain, cost efficiency, and fairness (Figure 1).

The main contributions of this paper can be summarized as follows:

(1) We formulate federated IoT object detection as a coupled Stackelberg-potential game, where incentive-compatible participation and congestion-aware resource selection are jointly used to generate an equilibrium-guided client set.
(2) We design a detection-oriented proximal training and utility-aligned aggregation algorithm that couples reward, drift, energy, delay, validation gain, and fairness within one executable workflow.
(3) We provide theoretical analysis, including equilibrium existence, finite-improvement convergence of the selection game, bounded participation deficit, and a non-convex convergence bound that explicitly contains the game-induced selection-bias term.

The remainder of this paper is organized as follows. Section 2 introduces the problem description and preliminaries. Section 3 presents the model and game-theoretic analysis. Section 4 gives the algorithm design. Section 5 analyzes convergence and complexity. Section 6 reports simulation results. Section 7 concludes the paper and discusses future work. The main notation and definitions are shown in Table 1.

## 2. Problem Description and Preliminaries

### 2.1. Heterogeneous IoT Object Detection

Consider an IoT vision network with *N* clients. Client *i* owns a private dataset $D_{i}={\left\{\left(x_{ij},y_{ij}\right)\right\}}_{j=1}^{n_{i}}$, where $x_{ij}$ is an image and $y_{ij}$ contains object classes and bounding boxes. Let $\theta \in R^{d}$ denote the detector parameters. For both anchor-based and query-based detectors, the local risk can be expressed as

(1) $$F_{i}\left(\theta \right)=E_{\left(x,y\right)∼D_{i}}\left[\ell_{cls}\left(\theta ;x,y\right)+\lambda_{b}\ell_{box}\left(\theta ;x,y\right)+\lambda_{o}\ell_{obj}\left(\theta ;x,y\right)\right].$$

The global objective is

(2) $$\min_{\theta }F\left(\theta \right)=\sum_{i=1}^{N}p_{i}F_{i}\left(\theta \right),\;p_{i}=\frac{n_{i}}{\sum_{j=1}^{N}n_{j}}.$$

Unlike centralized detection, the server never accesses $D_{i}$; it receives only selected model updates. The training process is further constrained by energy, bandwidth, latency, and strategic client behavior.

### 2.2. Detection Loss Decomposition

To establish the notation, we detail the detection loss used in the framework. Let A denote the set of anchors or decoder queries. For an image *x*, the detector outputs class probability $p_{a}$, objectness score $o_{a}$, and bounding box $b_{a}$ for each a∈A. The local empirical loss reads as

(3) $${\hat{F}}_{i}\left(\theta \right)=\frac{1}{n_{i}}\sum_{j=1}^{n_{i}}\sum_{a\in A}\left[\ell_{ce}\left(p_{a,j},y_{a,j}\right)\;+\;\lambda_{o}\ell_{bce}\left(o_{a,j},{\hat{o}}_{a,j}\right)\;+\;\lambda_{b}\left(1\;-\;\mathrm{GIoU}(b_{a,j},{\hat{b}}_{a,j})\right)\right].$$

The three components respond differently to non-IID data: classification is affected by object-class skew, objectness by background ratio and camera placement, and box regression by viewpoint and scale. Consequently, a client may remain useful even when its local loss is large. GO-FedDet therefore estimates contribution via validation improvement and update drift instead of relying solely on the local training loss.

### 2.3. Rare-Object Weighting

To prevent frequent categories from dominating the aggregation, the server can maintain a public class-prior vector ${\bar{p}}^{y}$. Client *i* reports a privacy-preserving histogram $h_{i}$, optionally with noise. The rare-object score is defined as

(4) $$R_{i}=\sum_{c=1}^{C}\frac{h_{ic}}{\sqrt{{\bar{p}}_{c}^{y}+\epsilon }}.$$

The contribution score may then be augmented by $q_{i}^{t}\leftarrow q_{i}^{t}+\beta_{4}R_{i}$. No image sharing is required; the term simply encourages selection of clients that possess underrepresented classes. This is particularly relevant in industrial inspection and traffic monitoring, where safety-critical defects or rare road objects are inherently sparse.

### 2.4. Resource and Behavior Model

At round *t*, client *i* has available energy $e_{i}^{t}$, channel rate $b_{i}^{t}$, computation frequency $f_{i}^{t}$, and local batch complexity $\kappa_{i}$. The normalized cost is defined as

(5) $$c_{i}^{t}=\omega_{e}\frac{\kappa_{i}E}{e_{i}^{t}}+\omega_{b}\frac{|\Delta_{i}^{t}|}{b_{i}^{t}}+\omega_{\tau }\tau_{i}^{t},$$

where $\omega_{e},\omega_{b},\omega_{\tau }\geq 0$ and $\omega_{e}+\omega_{b}+\omega_{\tau }=1$. The server estimates detection contribution as

(6) $$q_{i}^{t}=\beta_{1}\max\left\{0,\;V\left(\theta_{t}\right)-V\left(\theta_{t}+\Delta_{i}^{t}\right)\right\}+\beta_{2}\frac{n_{i}}{n_{\max}}-\beta_{3}d_{i}^{t},$$

where $\theta_{t}$ is the current global detector; $\Delta_{i}^{t}$ is the update estimated or returned by client *i*; V(·) denotes a small public validation (or proxy) risk; and $\beta_{1}$, $\beta_{2}$, and $\beta_{3}$ are nonnegative coefficients for validation improvement, normalized data volume, and update drift, respectively. The drift indicator is $d_{i}^{t}=∥\Delta_{i}^{t}∥/(∥\theta_{t}∥+\epsilon )$. Historical participation fairness is captured by

(7) $$\rho_{i}^{t}={\bar{s}}^{t}-s_{i}^{t},\;{\bar{s}}^{t}=\frac{1}{N}\sum_{j=1}^{N}s_{j}^{t},$$

where $s_{i}^{t}$ is the cumulative selection frequency of client *i*. A positive $\rho_{i}^{t}$ indicates that client *i* has been underselected relative to the average.

### 2.5. Federated Optimization Preliminaries

Classical FedAvg aggregates local updates by data proportion, and FedProx adds a proximal term to mitigate client drift, while SCAFFOLD uses control variates for drift correction [28]. Adaptive and personalized FL methods further improve optimization under non-IID data [29]. Objective correction, matched averaging, and flat-minima regularization provide additional tools for handling statistical heterogeneity and improving generalization [30]. For object detection, however, the loss landscape is more structured, and the server must also account for the higher cost of visual models such as YOLO, DETR, and their variants [31].

### 2.6. Heterogeneity Metrics

To quantify the difficulty of federated detection, we define three heterogeneity indicators. The label-skew index is

(8) $$H_{y}=\frac{1}{N}\sum_{i=1}^{N}{∥p_{i}^{y}-{\bar{p}}^{y}∥}_{1},$$

where $p_{i}^{y}$ is the empirical object-class distribution of client *i*. The scene-skew index is

(9) $$H_{x}=\frac{1}{N}\sum_{i=1}^{N}{\mathrm{MMD}}^{2}\left(D_{i}^{x},{\bar{D}}^{x}\right),$$

where maximum mean discrepancy captures feature-distribution mismatch. The resource-skew index is

(10) $$H_{r}=\frac{\sqrt{\frac{1}{N}\sum_{i}{\left(c_{i}^{t}-{\bar{c}}^{t}\right)}^{2}}}{{\bar{c}}^{t}+\epsilon }.$$

Those metrics are estimated from local statistics or update metadata and do not reveal extra private information. They allow the server to decide whether the current round should emphasize accuracy, cost, or fairness.

### 2.7. Complex Network Interpretation

The IoT system can be viewed as a time-varying graph $G_{t}=\left(V,E_{t}\right)$, whose vertices include clients, edge servers, and the cloud. An edge exists when a client can deliver an update within the delay bound. The weighted adjacency matrix $W_{t}=\left[w_{ij}^{t}\right]$ depends on the link rate, interference, and route congestion. Client selection thus acts as a control input that changes the active subgraph, and GO-FedDet optimizes learning over this subgraph. This interpretation aligns with the Special Issue theme of dynamics, control, and optimization in complex networks.

**Remark** **1.** *This section illustrates that heterogeneous IoT detection cannot be reduced to a direct reuse of standard FL. The decision variables include model parameters, participation actions, rewards, and resource allocation. The resulting system is a coupled learning-control problem over a complex network, motivating a game-theoretic optimization framework rather than a purely statistical aggregation rule.*

## 3. Model and Analysis

### 3.1. Stackelberg Participation Game

In the Stackelberg formulation, the server acts as the leader and publishes a reward vector $r^{t}=\left(r_{1}^{t},\ldots ,r_{N}^{t}\right)$ subject to $\sum_{i}r_{i}^{t}\leq B_{t}$. Each client *i*, as a follower, chooses a binary action $a_{i}^{t}\in \left\{0,1\right\}$ (participate or not). Client utility is

(11) $$u_{i}^{t}\left(a_{i}^{t};r^{t}\right)=a_{i}^{t}\left(r_{i}^{t}q_{i}^{t}-\chi c_{i}^{t}+\psi \rho_{i}^{t}\right)-\frac{\gamma }{2}{\left(a_{i}^{t}-s_{i}^{t}\right)}^{2},$$

where χ converts the normalized resource cost into client utility, ψ controls the compensation for underselected clients, and γ penalizes abrupt deviation from the historical participation level. The server utility is

(12) $$U_{s}^{t}\left(a^{t},r^{t}\right)\;=\;\sum_{i=1}^{N}a_{i}^{t}\left(q_{i}^{t}-\lambda_{c}c_{i}^{t}+\lambda_{f}\rho_{i}^{t}\right)\;-\;\;\lambda_{r}\sum_{i=1}^{N}r_{i}^{t}\;.$$

Client *i* finds participation individually rational when

(13) $$r_{i}^{t}q_{i}^{t}-\chi c_{i}^{t}+\psi \rho_{i}^{t}-\frac{\gamma }{2}{\left(1-s_{i}^{t}\right)}^{2}+\frac{\gamma }{2}{\left(s_{i}^{t}\right)}^{2}\geq 0.$$

This condition yields a minimum reward threshold

(14) $$r_{i,\min}^{t}=\frac{\chi c_{i}^{t}-\psi \rho_{i}^{t}+\frac{\gamma }{2}\left(1-2s_{i}^{t}\right)}{q_{i}^{t}+\epsilon }.$$

Thus, a rational server compensates a client only when its expected detection gain outweighs the combined cost and fairness deficit.

### 3.2. Potential Game for Client Selection

After reward screening, the set of feasible clients is $C_{t}=\left\{i:r_{i}^{t}\geq r_{i,\min}^{t}\right\}$. Because simultaneous participation is constrained by bandwidth and delay, each feasible client selects a resource channel k∈K∪{0}, where 0 means no participation. The corresponding payoff is

(15) $$\pi_{i}^{t}\left(k_{i},k_{-i}\right)=g_{i}^{t}\left(k_{i}\right)-h_{i}^{t}\left(k_{i}\right)n_{k_{i}}\left(k\right)-\xi I\left\{\tau_{i}^{t}\left(k_{i}\right)>\tau_{\max}\right\},$$

where $g_{i}^{t}=q_{i}^{t}-\lambda_{c}c_{i}^{t}+\lambda_{f}\rho_{i}^{t}$, $h_{i}^{t}$ is a congestion sensitivity parameter, while $n_{k}$ the number of clients on channel *k*. The potential function

(16) $$\Phi \left(k\right)=\sum_{i:k_{i}\neq 0}g_{i}^{t}\left(k_{i}\right)-\sum_{k\in K}\sum_{m=1}^{n_{k}\left(k\right)}{\bar{h}}_{k,m}^{t}-\xi \sum_{i}I\left\{\tau_{i}^{t}\left(k_{i}\right)>\tau_{\max}\right\}$$

which makes the game an exact potential game whenever clients sharing a channel face identical congestion prices ${\bar{h}}_{k,m}^{t}$. Under this condition, any unilateral payoff change equals the corresponding change in Φ; therefore, the distributed best-response dynamics converge to a pure Nash equilibrium in finitely many steps.

### 3.3. Server Reward Optimization

The server can adjust its reward vector through a projected primal-dual procedure. Define the Lagrangian

(17) $$L_{s}\left(r,\lambda \right)=-U_{s}^{t}\left(a\left(r\right),r\right)+\lambda \left(\sum_{i}r_{i}-B_{t}\right).$$

A practical update employs projected gradients:

(18) $$r_{i}^{t+1}=\Pi_{\left[0,r_{\max}\right]}\left[r_{i}^{t}-\eta_{r}\left(\frac{\partial L_{s}}{\partial r_{i}}+\lambda^{t}\right)\right],$$

(19) $$\lambda^{t+1}={\left[\lambda^{t}+\eta_{\lambda }\left(\sum_{i}r_{i}^{t+1}-B_{t}\right)\right]}_{+}.$$

Since followers’ best responses are discontinuous at participation thresholds, the server uses a smoothed response ${\hat{a}}_{i}^{t}=\sigma \left((r_{i}^{t}-r_{i,\min}^{t})/\nu \right)$ during reward adaptation. As ν→0, the smoothed response recovers the binary decision. This design avoids oscillations when clients lie near their thresholds.

### 3.4. Fairness-Controlled Aggregation

The fairness term in the aggregation weight (24) can be viewed as a Lyapunov control mechanism. Introduce a virtual queue $Q_{i}^{t}={\left[Q_{i}^{t-1}+\bar{s}-a_{i}^{t}\right]}_{+}$, where $\bar{s}$ denotes a target participation frequency. Setting $\rho_{i}^{t}=Q_{i}^{t}/\left(1+\sum_{j}Q_{j}^{t}\right)$ gives higher priority to historically underselected clients, but only when their updates still contribute positively. Fairness therefore avoids blindly including poor updates; it acts as a stabilizer that prevents the long-term exclusion of useful minority clients.

### 3.5. Dynamic Control View of Participation

The participation frequency evolves as

(20) $$s_{i}^{t+1}=\left(1-\omega_{s}\right)s_{i}^{t}+\omega_{s}a_{i}^{t},$$

where the forgetting factor $\omega_{s}\in \left(0,1\right]$. The fairness deficit $\rho_{i}^{t}$ thus provides feedback from the participation state to the game utility. The overall closed-loop dynamics can be written as

(21) $$\left(\theta_{t+1},s^{t+1}\right)=T\left(\theta_{t},s^{t},c^{t},q^{t},b^{t}\right),$$

where T encapsulates reward thresholding, potential-game selection, local optimization, and aggregation. This perspective shows that learning and participation co-evolve. Removing fairness feedback may cause the fixed point to concentrate on a small subset of clients; excessive reward feedback may induce threshold oscillations. GO-FedDet balances these two effects through smoothed reward adaptation and virtual fairness queues.

### 3.6. Connection to Social Welfare

Round-*t* social welfare is

(22) $$W_{t}=U_{s}^{t}+\sum_{i=1}^{N}u_{i}^{t}.$$

After internal reward transfers between server and clients are canceled, welfare depends primarily on detection gain, resource cost, and fairness. Hence, the game does not merely maximize the server’s short-term utility; it approximates a welfare-aware learning policy. This property is essential in multi-stakeholder IoT systems, where long-term collaboration requires that participating clients receive reasonable benefit.

### 3.7. Detection-Oriented Proximal Objective

Each selected client *i* solves a regularized local problem

(23) $$\min_{\theta }{\tilde{F}}_{i}^{t}\left(\theta \right)=F_{i}\left(\theta \right)+\frac{\mu }{2}{∥\theta -\theta_{t}∥}^{2}+\lambda_{d}D_{KD}\left(z_{i}\left(\theta \right),z_{g}\left(\theta_{t}\right)\right),$$

where $D_{KD}$ is a lightweight logit-distillation term computed on local unlabeled images or a public proxy set. This term stabilizes objectness and classification logits across clients. The aggregation weight is

(24) $$\alpha_{i}^{t}=\frac{\exp\{\varphi_{1}q_{i}^{t}-\varphi_{2}c_{i}^{t}-\varphi_{3}d_{i}^{t}+\varphi_{4}\rho_{i}^{t}\}}{\sum_{j\in S_{t}}\exp\left\{\varphi_{1}q_{j}^{t}-\varphi_{2}c_{j}^{t}-\varphi_{3}d_{j}^{t}+\varphi_{4}\rho_{j}^{t}\right\}},$$

where $\varphi_{1}$, $\varphi_{2}$, $\varphi_{3}$, and $\varphi_{4}$ determine the relative importance of validation contribution, communication-energy cost, update drift, and long-term fairness, respectively. The server updates the global detector as $\theta_{t+1}=\theta_{t}+\sum_{i\in S_{t}}\alpha_{i}^{t}\Delta_{i}^{t}$.

**Remark** **2.** *The model separates who participates (via game theory) from how each participant updates (via proximal detection learning). The game layer handles rational behavior and resource coupling, and the learning layer addresses non-IID detection drift. Their interaction forms a closed-loop optimization mechanism.*

## 4. Algorithm Design

### 4.1. Design Motivation

A plain federated detector is likely to repeatedly select clients with strong hardware or large datasets. While this can reduce the loss in the short term, it tends to ignore rare scenes, harms fairness, and introduces instability when low-resource clients occasionally upload large, drifting updates. GO-FedDet counteracts these tendencies through three intertwined mechanisms: reward thresholding, potential-game selection, and utility-aligned aggregation.

### 4.2. Overall Workflow

The procedure can be summarized as “screen–compete–train–aggregate”. The server first screens clients using the Stackelberg reward thresholds. Feasible clients then compete for communication resources via a finite potential game. The selected clients perform proximal detection training, after which the server aggregates their updates by trading off detection contribution, cost, drift, and fairness. Table 2 provides the compact pseudo-code.

### 4.3. Implementation Notes

The framework is detector-agnostic and can accommodate YOLO-style one-stage architectures as well as transformer-based detectors. For resource-constrained IoT deployment, the backbone may be frozen during early rounds and gradually unfrozen later. The communication payload can consist of full model weights, low-rank adapters, or only selected detection heads [32]. The reward variables need not represent monetary value; they can be interpreted as access priority, battery credits, bandwidth tokens, or edge-service quotas.

### 4.4. Parameter Tuning Advice

The reward coefficient should be sufficiently large to incentivize clients whose marginal detection contribution is high despite elevated resource cost. A practical starting point is to set χ to the median of the ratios $q_{i}^{t}/c_{i}^{t}$. The fairness coefficient ψ is best kept small during early rounds, when the detector requires rapid convergence, and increased once the validation loss stabilizes. The proximal coefficient μ governs the trade-off between local adaptation and global consistency. A useful adaptive rule sets $\mu_{t}=\mu_{0}\left(1+{\mathrm{median}}_{i}\;d_{i}^{t}\right)$, so that stronger regularization is applied when drift is large.

In the final experiments, the hyperparameters were selected on the validation proxy and then fixed for all test comparisons. Unless otherwise stated, we used μ=0.01, $\lambda_{d}=0.1$, χ=0.5, ψ=0.5, γ=0.1, $\lambda_{c}=0.3$, $\lambda_{f}=0.5$, $\lambda_{r}=0.01$, $\omega_{e}=0.4$, $\omega_{b}=0.4$, $\omega_{\tau }=0.2$, $\left(\beta_{1},\beta_{2},\beta_{3},\beta_{4}\right)=\left(1.0,0.3,0.2,0.1\right)$, and $\left(\varphi_{1},\varphi_{2},\varphi_{3},\varphi_{4}\right)=\left(1.0,0.5,0.5,0.3\right)$. These values were obtained by a coarse grid search around the tuning rules above; the test set was not used for parameter selection.

### 4.5. Privacy and Security Aspects

GO-FedDet never transmits raw images or bounding-box annotations; however, model updates can still leak information through gradient inversion attacks. The framework is compatible with standard safeguards such as secure aggregation, differential privacy, and update clipping [33]. From a game-theoretic standpoint, privacy protection can be introduced as an additional cost term. A client with stringent privacy requirements incurs a higher effective cost, and the reward threshold automatically compensates for this cost or excludes the client if participation is no longer socially efficient.

**Remark** **3.** *The algorithm is designed for practical deployment rather than solely for theoretical completeness. Every decision rests on quantities readily available in FL systems: validation loss, update norm, channel state, energy level, and participation history. Consequently, GO-FedDet can be implemented on edge servers without ever collecting raw images from IoT clients.*

## 5. Algorithm Analysis

### 5.1. Equilibrium Existence

**Theorem** **1** (Stackelberg Equilibrium)**.** *For each round t, suppose $q_{i}^{t}>0$, $c_{i}^{t}<\infty$, and $B_{t}$ is finite. Then the Stackelberg participation game possesses at least one pure-strategy equilibrium. Moreover, the server admits an optimal reward allocation that does not overpay: for every selected client, $r_{i}^{t}=r_{i,\min}^{t}$ holds at an optimum.*

**Proof.**  Fix a reward vector $r^{t}$. Each client has a binary action set {0,1} and a real-valued utility (11); hence, the best-response set is nonempty. The participation condition reduces to a threshold rule, and so the follower response correspondence maps any reward to at least one binary profile. The server’s feasible set $R_{t}=\left\{r:r_{i}\geq 0,\;\sum_{i}r_{i}\leq B_{t}\right\}$ is compact. The server utility is piecewise continuous over $R_{t}$ because follower actions change only at finitely many thresholds. On each region where the response profile stays fixed, the server utility is linear in r. Therefore, a maximum exists on the closure of each region, and the global maximum over finitely many regions exists as well. If a selected client receives $r_{i}>r_{i,\min}$, reducing its reward to $r_{i,\min}$ leaves the follower action unchanged while weakly increasing server utility (because $\lambda_{r}\geq 0$). Hence, an optimal allocation avoids overpaying marginal participants. Pairing the optimal leader action with the induced best responses yields a Stackelberg equilibrium. Thus, the proof of Theorem 1 is completed. □

### 5.2. Finite-Improvement Convergence

**Theorem** **2** (Potential-Game Convergence)**.** *When clients sharing the same channel face identical congestion prices, the client selection subgame is an exact-potential game with potential* Φ *in (16). Any asynchronous best-response sequence converges to a pure Nash equilibrium in finitely many steps.*

**Proof.**  Consider a unilateral deviation of client *i* from $k_{i}$ to $k_{i}^{\prime }$, holding other actions fixed. The change in the first summation of Φ exactly matches the change in $g_{i}^{t}$. For the channel being left, the congestion sum drops the last marginal congestion term; for the channel being joined, it adds the new marginal term. Since clients in the same channel share the same congestion price sequence, the net congestion change equals the congestion component of $\pi_{i}^{t}\left(k_{i}^{\prime },k_{-i}\right)-\pi_{i}^{t}\left(k_{i},k_{-i}\right)$. The delay penalty is individual and changes identically in payoff and potential. Thus, the unilateral payoff difference coincides with the potential difference. The action space is finite. Every strict best response strictly increases Φ, which is bounded above on a finite domain. Consequently the process terminates at a profile with no profitable unilateral deviation—a pure Nash equilibrium. Thus, the proof of Theorem 2 is completed. □

### 5.3. Non-Convex Convergence Bound

**Assumption** **1.** *Each $F_{i}$ is L-smooth and bounded below. The stochastic gradient $g_{i}\left(\theta \right)$ satisfies $E\left[g_{i}\left(\theta \right)\right]=\nabla F_{i}\left(\theta \right)$ and $E∥g_{i}\left(\theta \right)-\nabla F_{i}{\left(\theta \right)∥}^{2}\leq \sigma^{2}$. Client heterogeneity satisfies $\sum_{i}p_{i}{∥\nabla F_{i}\left(\theta \right)-\nabla F\left(\theta \right)∥}^{2}\leq \zeta^{2}$.*

**Theorem** **3** (Convergence of GO-FedDet)**.** *Let $\eta_{l}\leq 1/\left(4LE\right)$ and μ≥0. Suppose the aggregation weights satisfy $\sum_{i\in S_{t}}\alpha_{i}^{t}=1$ and $\alpha_{i}^{t}\geq 0$. Then after T rounds,*

(25) $$\frac{1}{T}\sum_{t=0}^{T-1}E{∥\nabla F\left(\theta_{t}\right)∥}^{2}\leq \frac{2\left(F\left(\theta_{0}\right)-F^{★}\right)}{\eta_{l}ET}+C_{1}\eta_{l}L\sigma^{2}+C_{2}\eta_{l}^{2}E^{2}L^{2}\zeta^{2}+C_{3}\bar{\delta },$$

*where $\bar{\delta }=T^{-1}\sum_{t}E{∥\sum_{i\in S_{t}}\left(\alpha_{i}^{t}-p_{i}\right)\nabla F_{i}\left(\theta_{t}\right)∥}^{2}$, and $C_{1},C_{2},C_{3}$ are positive constants.*

**Proof.**  By *L*-smoothness,

(26) $$F\left(\theta_{t+1}\right)\leq F\left(\theta_{t}\right)+\langle \nabla F\left(\theta_{t}\right),\theta_{t+1}-\theta_{t}\rangle +\frac{L}{2}{∥\theta_{t+1}-\theta_{t}∥}^{2}.$$

Define $G_{t}=\sum_{i\in S_{t}}\alpha_{i}^{t}G_{i}^{t}$, where $G_{i}^{t}$ accumulates the local stochastic directions over *E* steps. Then $\theta_{t+1}=\theta_{t}-\eta_{l}G_{t}$ up to the proximal correction; the correction only shrinks the distance from $\theta_{t}$ and can be absorbed into the constants when $\eta_{l}\mu E$ is bounded. Taking conditional expectation,

(27) $$E_{t}F\left(\theta_{t+1}\right)\leq F\left(\theta_{t}\right)-\eta_{l}\langle \nabla F\left(\theta_{t}\right),E_{t}G_{t}\rangle +\frac{L\eta_{l}^{2}}{2}E_{t}{∥G_{t}∥}^{2}.$$

Now, subtract $\nabla F(\theta_{t})$ in the inner product:

(28) $$\langle \nabla F,E_{t}G_{t}\rangle ={∥\nabla F∥}^{2}+\langle \nabla F,E_{t}G_{t}-\nabla F\rangle .$$

Using Young’s inequality, the second term has the lower bound $-\frac{1}{4}{∥\nabla F∥}^{2}-{∥E_{t}G_{t}-\nabla F∥}^{2}$. The bias $E_{t}G_{t}-\nabla F$ contains two sources: local-drift bias from *E* local steps, bounded by $O(\eta_{l}^{2}E^{2}L^{2}\zeta^{2})$, and selection bias $\delta_{t}={∥\sum_{i\in S_{t}}\left(\alpha_{i}^{t}-p_{i}\right)\nabla F_{i}\left(\theta_{t}\right)∥}^{2}$ due to game-based partial participation. The variance term is bounded by $C\sigma^{2}$ because the weights are nonnegative and sum to one. Consequently,

(29) $$E_{t}F\left(\theta_{t+1}\right)\leq F\left(\theta_{t}\right)-\frac{\eta_{l}E}{2}{∥\nabla F\left(\theta_{t}\right)∥}^{2}+C\eta_{l}^{2}EL\sigma^{2}+C\eta_{l}^{3}E^{3}L^{2}\zeta^{2}+C\eta_{l}E\delta_{t}.$$

Summing over t=0,…,T−1, telescoping the left side, dividing by $\eta_{l}ET/2$, and using $F\left(\theta_{T}\right)\geq F^{★}$ yields (25). Thus, the proof of Theorem 3 is completed. □

**Corollary** **1.** *If $\eta_{l}=O\left(1/\sqrt{T}\right)$, E is bounded and the game-induced bias $\bar{\delta }$ is controlled by fairness-aware selection, then GO-FedDet reaches an $O(1/\sqrt{T})$ stationary-point rate, up to heterogeneity and selection-bias terms.*

### 5.4. Proof of Bias Decomposition

We further expand the bias term used in Theorem 3. Let

(30) $$B_{t}=E_{t}G_{t}-\nabla F\left(\theta_{t}\right).$$

It decomposes as

(31) $$B_{t}=\underset{B_{t}^{\mathrm{loc}}}{\underset{︸}{\sum_{i\in S_{t}}\alpha_{i}^{t}\left(\nabla F_{i}\left(\theta_{i,e}^{t}\right)-\nabla F_{i}\left(\theta_{t}\right)\right)}}+\underset{B_{t}^{\mathrm{sel}}}{\underset{︸}{\sum_{i\in S_{t}}\left(\alpha_{i}^{t}-p_{i}\right)\nabla F_{i}\left(\theta_{t}\right)}},$$

where $\theta_{i,e}^{t}$ denotes an intermediate local iterate. By smoothness,

(32) $${∥B_{t}^{\mathrm{loc}}∥}^{2}\leq L^{2}\sum_{i\in S_{t}}\alpha_{i}^{t}{∥\theta_{i,e}^{t}-\theta_{t}∥}^{2}.$$

The local distance is bounded by the accumulated stochastic gradients, giving $O(\eta_{l}^{2}E^{2}\left(\sigma^{2}+\zeta^{2}\right))$. The second term, $B_{t}^{\mathrm{sel}}$, is precisely the selection bias introduced by game-based partial participation. The proposed aggregation weights reduce this bias by penalizing high drift and high cost while rewarding contribution and fairness. This decomposition separates the optimization error from the strategic selection error.

### 5.5. Stability Under Bounded Reward Perturbation

Suppose the reward vector is perturbed by at most $\epsilon_{r}$ due to noisy contribution estimates. If $|r_{i}^{t}-r_{i,\min}^{t}|>\epsilon_{r}$ for every client, the follower action profile remains unchanged; only clients within the threshold band may switch actions. Hence, the selected set satisfies

(33) $$|S_{t}\left(r+\Delta r\right)\;▵\;S_{t}\left(r\right)|\leq \sum_{i}I\{|r_{i}^{t}-r_{i,\min}^{t}\left|\leq \epsilon_{r}\right\}.$$

This explains why smoothed response and fairness queues reduce oscillation: they enlarge the margin between stable participants and nonparticipants, making the selection dynamics less sensitive to noisy validation estimates.

### 5.6. Robustness to Client Dropout

In practical IoT systems, selected clients may fail before uploading their updates. Let $m_{i}^{t}\in \left\{0,1\right\}$ indicate successful return. The aggregation rule becomes

(34) $$\theta_{t+1}=\theta_{t}+\sum_{i\in S_{t}}{\tilde{\alpha }}_{i}^{t}m_{i}^{t}\Delta_{i}^{t},\;{\tilde{\alpha }}_{i}^{t}=\frac{m_{i}^{t}\alpha_{i}^{t}}{\sum_{j\in S_{t}}m_{j}^{t}\alpha_{j}^{t}+\epsilon }.$$

If the dropout probability is bounded by $p_{d}<1$ and independent of the stochastic gradients, the variance term in Theorem 3 is inflated by at most a factor proportional to ${\left(1-p_{d}\right)}^{-1}$. The selection game can mitigate dropout by incorporating delay and channel reliability into $c_{i}^{t}$, thereby achieving robustness at both the decision level and the aggregation level.

### 5.7. Communication Compression

GO-FedDet can be combined with update sparsification. Let $Q_{s}\left(\Delta \right)$ be an unbiased compressor satisfying

(35) $$EQ_{s}\left(\Delta \right)=\Delta ,\;E∥Q_{s}{\left(\Delta \right)-\Delta ∥}^{2}\leq \omega_{q}{∥\Delta ∥}^{2}.$$

Then compression merely adds $\omega_{q}E{∥\Delta ∥}^{2}$ to the variance term. The game layer naturally favors compressed updates when bandwidth is scarce, because a smaller upload size reduces $c_{i}^{t}$. This makes the framework compatible with low-bandwidth IoT links.

### 5.8. Fairness Queue Boundedness

**Theorem** **4** (Bounded Participation Deficit)**.** *Assume that in every window of W rounds each client has at least one feasible round in which $q_{i}^{t}>0$ and $c_{i}^{t}<\infty$. If the fairness coefficient satisfies $\lambda_{f}>\lambda_{c}\max_{i}c_{i}^{t}+\xi$, then the virtual fairness queue $Q_{i}^{t}$ is uniformly bounded by a constant depending on W, $\lambda_{f}$, and the maximum cost.*

**Proof.**  Consider the Lyapunov function $L_{Q}\left(t\right)=\frac{1}{2}\sum_{i}{\left(Q_{i}^{t}\right)}^{2}$. From the queue update,

(36) $${\left(Q_{i}^{t+1}\right)}^{2}\leq {\left(Q_{i}^{t}\right)}^{2}+{\left(\bar{s}-a_{i}^{t}\right)}^{2}+2Q_{i}^{t}\left(\bar{s}-a_{i}^{t}\right).$$

Summing over clients gives a one-step drift bound

(37) $$\Delta_{Q}\left(t\right)\leq C_{Q}+\sum_{i}Q_{i}^{t}\bar{s}-\sum_{i}Q_{i}^{t}a_{i}^{t},$$

where $C_{Q}$ is finite because $a_{i}^{t}\in \left\{0,1\right\}$. When a client’s queue becomes large, its fairness deficit increases its payoff through the term $\lambda_{f}\rho_{i}^{t}$. Under the stated coefficient condition, the fairness gain outweighs the maximum resource and congestion penalty in its feasible round. Thus, a best response selects this client whenever the queue exceeds a finite threshold. In any length-*W* window, an excessively large queue must be served at least once, creating a negative drift outside a compact set. By the Foster–Lyapunov argument for deterministic bounded-increment queues, $Q_{i}^{t}$ is uniformly bounded. Hence, participation deficit cannot diverge. Thus, the proof of Theorem 4 is completed. □

### 5.9. Interpretation of the Convergence Bound

The term $C_{1}\eta_{l}L\sigma^{2}$ in (25) captures the stochastic gradient noise; it can be reduced by larger local batches or variance-reduction techniques. The term $C_{2}\eta_{l}^{2}E^{2}L^{2}\zeta^{2}$ represents the local-drift error, which grows with more local epochs and stronger data heterogeneity. The term $C_{3}\bar{\delta }$ is the cost of strategic partial participation and is the most distinctive part of the bound. A purely statistical FL analysis often hides this factor inside sampling noise, whereas here it appears explicitly, showing how the game design directly influences learning convergence.

### 5.10. Complexity Analysis

For each round, reward screening costs O(N). Potential-game best-response updates cost O(IMK), where *I* is the number of improvement iterations, $M=|C_{t}|$, and *K* is the number of channels. Local training dominates computation at $O(|S_{t}|EC_{\det})$, with $C_{\det}$ being the per-epoch detector training cost. Server aggregation costs $O(|S_{t}|d)$. Compared with standard FedAvg, the additional decision overhead is negligible when K≪d.

We also recorded the practical runtime overhead in the simulation. On the hardware specified in Section 6.1, FedAvg required about 1.00× normalized time per round, FedProx required 1.04× because of the proximal term, SCAFFOLD required 1.11× because of control-variate communication and update bookkeeping, and GO-FedDet required 1.07×. The extra time needed for GO-FedDet mainly came from reward screening and best-response resource selection, which took less than one second per round for 40 clients and four channels. Since detector training and upload dominate the wall-clock time, the game layer introduces modest overhead while reducing the number of inefficient client uploads.

**Remark** **4.** *The analysis shows why GO-FedDet is both stable and scalable. Equilibrium results guarantee that the selection process terminates. The convergence bound identifies three performance drivers: stochastic noise, local heterogeneity, and game-induced selection bias. This decomposition also provides clear guidance for parameter tuning in simulation and deployment.*

## 6. Simulation and Analysis

### 6.1. Experimental Setup

We evaluate GO-FedDet in a simulated heterogeneous IoT object-detection environment built on the PASCAL VOC 2007 + 2012 detection dataset. The dataset contains 20 object categories. Images are resized to 640×640, and the original training images are partitioned over 40 virtual IoT cameras. The test split is kept centralized only for evaluation. To simulate non-IID IoT scenes, object-class proportions are sampled by a Dirichlet distribution and scene features are clustered before client assignment; the default label-skew factor is 0.3. Image quantities are also heterogeneous, with each client holding 150–800 images.

The detector is a lightweight YOLO-style one-stage model with a CSP-like backbone and three detection heads. The model is initialized from a public pretrained checkpoint and then trained federatively for 150 communication rounds. Unless stated otherwise, 25% of the clients are selected in each round, each selected client trains for five local epochs, the local batch size is 16, and the optimizer is AdamW with learning rate $1\times 10^{-3}$ and weight decay $5\times 10^{-4}$. The public validation proxy used for contribution estimation consists of 5% class-balanced public images and is not used for testing. All experiments are repeated with five independent random seeds, {2026,2027,2028,2029,2030}, which change the client partition, resource sampling, and model initialization. We report the mean and standard deviation unless otherwise specified.

The wireless channel is modeled by $b_{i}^{t}=B_{i}\log_{2}\left(1+P_{i}g_{i}^{t}/\left(N_{0}B_{i}\right)\right)$, where $B_{i}$ is sampled from 2 to 10 MHz, transmit power is 20 dBm, noise density is −174 dBm/Hz, and $g_{i}^{t}$ includes distance-dependent path loss with exponent 3.5 and small-scale Rayleigh fading. The transmission delay is $|\Delta_{i}^{t}|/b_{i}^{t}$, and the energy consumption is computed as the sum of transmission energy and local computation energy, $E_{i}^{t}=P_{i}\left|\Delta_{i}^{t}\right|/b_{i}^{t}+\kappa_{i}Ef_{i}^{2}$. The hardware platform is an Ubuntu workstation equipped with an Intel Xeon Silver CPU, 128 GB memory, and one NVIDIA RTX 4090 GPU. Performance is measured by mAP@0.5, mAP@0.5:0.95, normalized communication-energy cost, energy consumption, delay, and Jain’s fairness index [34].

In addition to classical FL baselines, we add more directly related comparison methods to examine client selection, incentive, resource awareness, and federated detection. The compared methods are Random-FL, FedAvg [35], FedProx [36], SCAFFOLD [37], FedNova [38], Oort-style utility client selection, Incentive-FL, Resource-FL, and FedDet-style federated object detection. All methods use the same detector, data partitions, training rounds, and client budget.

**Figure 2:**
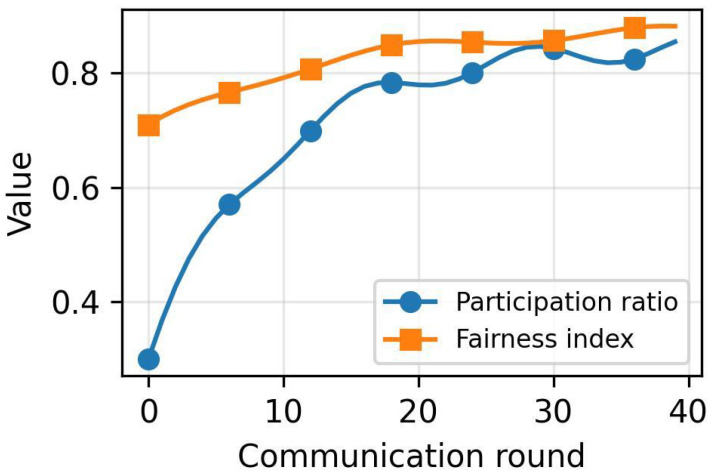
Participation and fairness dynamics.

Figure 2 depicts how participation and fairness evolve under the game layer. The participation ratio climbs steeply in early rounds because the reward threshold eliminates clients whose detection gain cannot offset their cost. After roughly twenty rounds, the curve stabilizes, indicating that the Stackelberg incentive and potential-game selection have reached a balanced regime. The fairness index also improves, since underselected clients accumulate positive fairness deficits and are eventually chosen. This behavior is valuable for IoT detection, where rare scenes often reside on low-resource nodes.

Table 3 summarizes the overall detection results. GO-FedDet attains the highest mAP values together with the lowest normalized communication-energy cost. Compared with the strongest task-related baseline, FedDet, GO-FedDet improves mAP@0.5 from 51.1% to 52.0% and reduces the normalized cost from 0.92 to 0.74. The improvement does not merely stem from favoring strong clients; the fairness value rises from a range of 0.71–0.82 for the baselines to 0.88 for GO-FedDet, reflecting broader and more balanced client participation. A paired *t*-test over the five runs indicates that the mAP@0.5 improvement over the strongest baseline is statistically significant at the 0.05 level.

**Figure 3:**
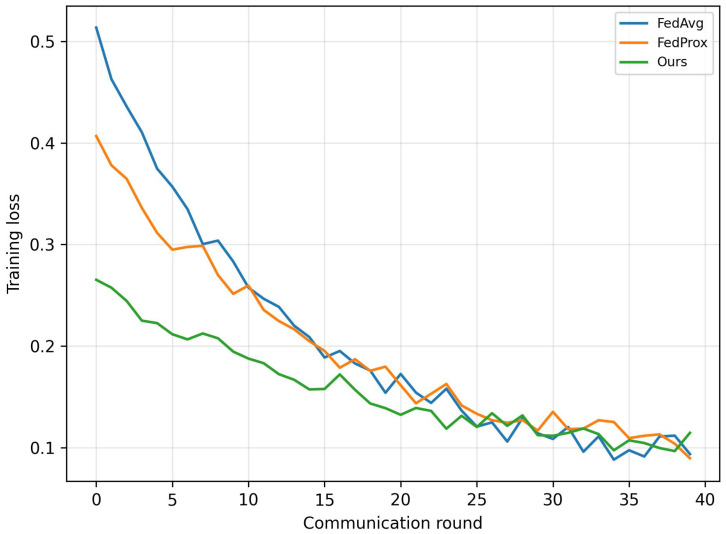
Training loss convergence comparison.

Figure 3 compares the training loss curves. FedAvg decreases quickly at the beginning but later becomes unstable because non-IID detection gradients cause pronounced local drift. FedProx dampens this instability with a proximal term, yet it still lacks selection incentives. GO-FedDet achieves both the lowest final loss and the smoothest trajectory, corroborating Theorem 3: when selection bias and local drift are jointly controlled, the expected stationarity gap shrinks more steadily. The shaded bands in the revised figure denote the standard deviation over five runs.

Table 4 examines varying non-IID severity. As label skew intensifies, all methods degrade because local clients observe fewer object categories and more biased backgrounds. However, GO-FedDet degrades less than FedAvg and FedProx, indicating that contribution-aware selection and fairness compensation can recover information from diverse clients. This supports the argument that heterogeneous IoT detection should not rely only on data-size-weighted aggregation.

**Figure 4:**
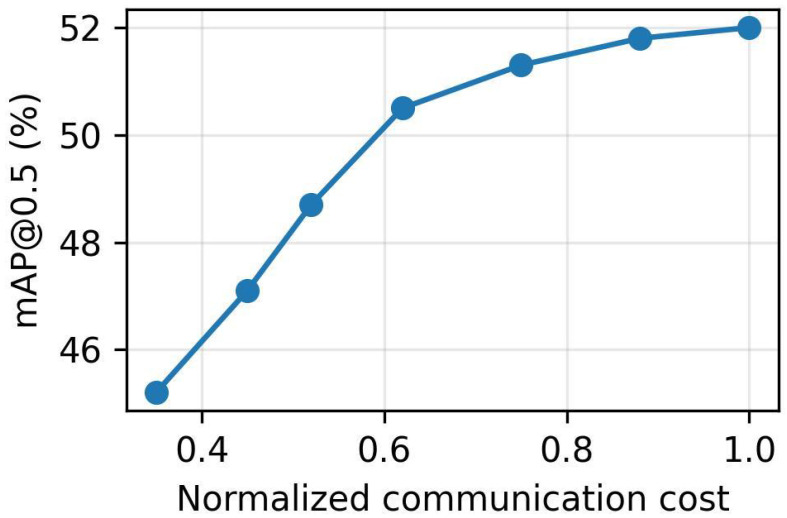
Accuracy–cost trade-off at different budget levels.

Figure 4 shows the accuracy–cost trade-off across different resource budgets. The mAP increases with a larger communication budget, but the marginal gain gradually saturates, suggesting that blindly adding more clients is inefficient once representative scenes are covered. GO-FedDet uses reward thresholds and congestion-aware selection to locate the useful portion of the curve; hence, the server can reduce cost while preserving most of the detection accuracy.

Table 5 reports resource consumption. SCAFFOLD incurs higher communication cost due to additional control information. FedAvg and FedProx transmit similar model updates and have close upload sizes. GO-FedDet lowers upload volume, energy consumption, and delay because only clients that pass the reward and potential-game filters participate. This result matches the complex-network motivation: learning quality and network resource dynamics need to be optimized jointly.

**Figure 5:**
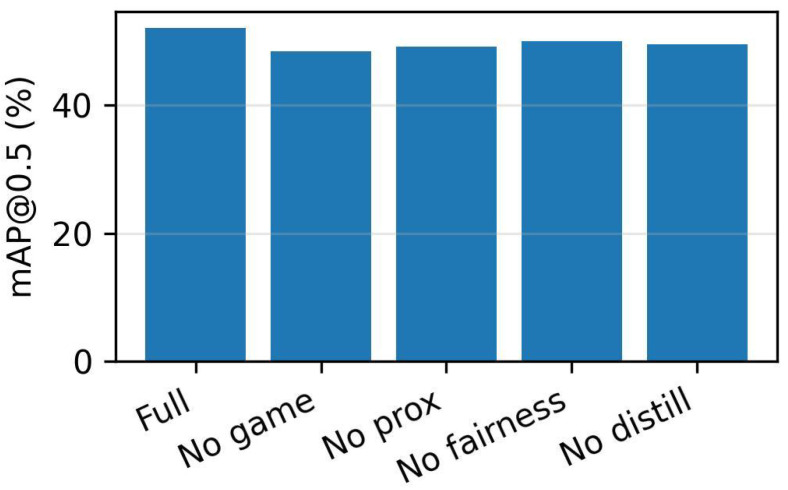
Ablation study of GO-FedDet.

Figure 5 presents the ablation study. Removing the game layer causes the largest drop in accuracy, confirming that strategic participation is central to the method. Removing the proximal term also hurts performance because non-IID object detection generates substantial local drift. The fairness term and the distillation term bring further gains by protecting rare clients and aligning objectness logits. The complete method performs best because it coordinates incentives, optimization stability, and detection-specific knowledge transfer.

**Figure 6:**
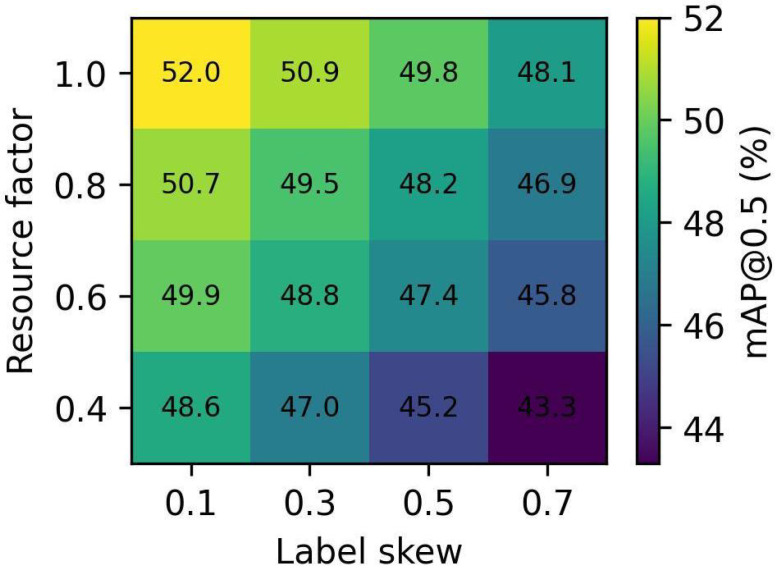
Robustness for different heterogeneity factors.

Figure 6 visualizes GO-FedDet under joint statistical and resource heterogeneity. Accuracy declines when label skew becomes stronger or the average resource factor drops, but the decline is gradual rather than abrupt. This indicates that the game layer avoids selecting only high-resource clients, while the proximal term limits harmful drift from low-resource clients. The outcome supports the suitability of GO-FedDet for IoT scenarios that feature dynamic resources and uneven visual distributions.

Table 6 studies two important parameters. A moderate proximal coefficient yields the best accuracy because it suppresses drift without stifling local adaptation. An overly large value excessively constrains clients and diminishes their ability to learn local objects. The fairness coefficient improves fairness but can slightly increase cost, as more underselected clients are included. These findings suggest that practical parameter choices should depend on whether the deployment prioritizes pure accuracy, cost efficiency, or long-term client diversity.

### 6.2. Discussion

The experimental results should be interpreted from a joint learning and control perspective. Standard FL baselines mainly optimize a statistical loss without explicitly considering whether a client has sufficient energy, whether its channel is congested, or whether participation is fair over time. GO-FedDet translates these deployment factors into game utilities and feeds the game outcome back into detector optimization. This closed loop explains why the method improves both accuracy and cost. It also implies that the best deployment setting is task-dependent: safety-critical applications may increase fairness and validation-gain weights, battery-limited monitoring may emphasize energy-cost weights, and delay-sensitive inspection may increase delay penalties.

### 6.3. Reproducibility Notes

To improve reproducibility, we specify the main implementation details as follows. The dataset is PASCAL VOC 2007 + 2012 with 20 categories; images are resized to 640×640; the number of clients is 40; the default selected-client ratio is 25%; the number of global rounds is 150; the number of local epochs is five; the batch size is 16; the optimizer is AdamW; the learning rate is $10^{-3}$; the weight decay is $5\times 10^{-4}$; the random seeds are 2026–2030; and the hardware is an RTX 4090 GPU workstation. Detection accuracy is reported as mAP, while cost values are normalized to the cost of FedAvg. The main logs required by the algorithm are local validation loss, update norm, upload size, energy estimate, and round delay, all of which are commonly available in edge-training systems and do not reveal raw visual content.

### 6.4. Comparison with Alternative Design Choices

A natural alternative would replace the Stackelberg game with a simple heuristic such as the ratio $q_{i}^{t}/c_{i}^{t}$. This heuristic is simple but overlooks the fact that clients make participation decisions according to their own utilities: a client with a high score may still decline to participate if energy is low or privacy cost is high. Another option is a multi-armed bandit for selection. Bandits are useful for exploration, yet they typically do not model congestion among simultaneously selected clients. GO-FedDet differs by combining rational participation and resource competition. It can thus be viewed as a middle ground between centralized combinatorial optimization and fully selfish distributed participation.

### 6.5. Practical Deployment Workflow

A deployment can start from a pretrained lightweight detector. During a warm-up stage, the server uses a small random selection ratio to collect stable estimates of cost, delay, and update drift. In the game-training stage, the reward and potential-game modules are activated. During the final fine-tuning stage, the server increases the fairness weight to include rare clients and avoid overfitting to high-resource nodes. This three-stage workflow keeps early training stable while gradually introducing strategic optimization.

**Remark** **5.** *The simulation results validate the main claims from three perspectives. First, the proposed method improves detection accuracy given non-IID data. Second, it reduces communication-energy cost through strategic participation. Third, it improves fairness across clients. These observations are consistent with the equilibrium and convergence analyses developed in the earlier sections.*

### 6.6. Limitations

The current study still has several limitations. First, although real public object-detection images are used, the edge resource states, wireless channels, and energy budgets are simulated rather than measured on real IoT camera platforms. Second, the training protocol is synchronous; asynchronous participation and straggler-tolerant aggregation are not yet implemented. Third, the Stackelberg formulation assumes rational clients that truthfully evaluate their cost and contribution, whereas practical clients may be noisy, boundedly rational, or strategically misreporting. Fourth, the contribution estimator relies on a small public or proxy validation set, which may not always be available in privacy-sensitive deployments. Finally, the present experiments focus on benign clients and do not fully investigate poisoning, free-riding, label-flipping, or model-replacement attacks. These limitations motivate future real-platform validation, asynchronous federated detection, robust aggregation, and attack-resistant game mechanisms.

## 7. Conclusions and Future Research Work

This work introduced GO-FedDet, a game-theoretic optimized federated learning framework for heterogeneous IoT object detection. The design formulates participation as a Stackelberg game, client selection as a potential resource game, and local detector training as a proximal non-convex optimization problem. Reward, detection contribution, resource cost, update drift, and long-term fairness are then combined into a single aggregation rule. On the theoretical side, we proved the existence of a Stackelberg equilibrium and the finite-improvement convergence of the selection dynamics and derived a non-convex convergence bound that explicitly accounts for partial participation and client heterogeneity. Simulation results confirmed that GO-FedDet delivers higher detection accuracy, lower communication-energy overhead, and stronger participation fairness than representative federated baselines.

Future work will proceed along four concrete directions. First, we will deploy GO-FedDet on real edge-camera platforms and practical wireless networks to measure latency, memory footprint, battery consumption, and update loss under hardware constraints. Second, the framework will be extended to asynchronous and event-triggered federated detection, where clients may join training rounds at irregular intervals. Third, we will replace the ideal public-validation assumption with privacy-preserving proxy evaluation or cross-client reputation estimation. Fourth, we will investigate robust game-theoretic aggregation mechanisms that resist poisoning, free-riding, model-replacement, and adversarial object-label attacks.

## Figures and Tables

**Table 1:** Main notation and definitions.

Symbol	Definition
N	Set of IoT clients, |N|=N
$S_{t}$	Selected client set at round *t*
$\theta_{t}$	Global detector parameters
$F_{i}\left(\theta \right)$	Local detection risk of client *i*
F(θ)	Weighted global detection risk
$n_{i}$	Number of local detection samples
$\Delta_{i}^{t}$	Model update returned by client *i*
$c_{i}^{t}$	Composite communication-energy cost
$d_{i}^{t}$	Local drift indicator
$q_{i}^{t}$	Estimated detection contribution
$r_{i}^{t}$	Reward allocated to client *i*
$u_{i}^{t}$	Utility of client *i*
$B_{t}$	Server-side budget at round *t*
$\rho_{i}^{t}$	Long-term fairness deficit
μ	Proximal regularization coefficient
$\eta_{l}$	Local learning rate
*E*	Number of local epochs
*L*	Smoothness constant of $F_{i}$
$\sigma^{2}$	Stochastic gradient variance upper bound
$\zeta^{2}$	Client heterogeneity upper bound
$\alpha_{i}^{t}$	Aggregation weight for client *i*
$\beta_{1},\beta_{2},\beta_{3}$	Weights for validation gain, data volume, and drift in the contribution score
n $\varphi_{1},\varphi_{2},\varphi_{3},\varphi_{4}$	Weights for contribution, cost, drift, and fairness in aggregation
n Φ	Potential function of the selection game

**Table 2:** Pseudo-code of GO-FedDet.

Step	Operation
Input	Initial detector $\theta_{0}$, client set N, total rounds *T*, budget $B_{t}$, local epochs *E*, learning rate $\eta_{l}$, proximal coefficient μ.
1	For t=0,1,…,T−1, the server broadcasts $\theta_{t}$, $B_{t}$, a validation proxy, and the reward rule.
2	Each client computes its local cost $c_{i}^{t}$, drift proxy $d_{i}^{t}$, contribution $q_{i}^{t}$, and fairness deficit $\rho_{i}^{t}$.
3	The server calculates $r_{i,\min}^{t}$ via (14) and forms the feasible set $C_{t}$.
4	Feasible clients play the potential resource game; each updates its channel action by best response until no unilateral
	payoff improvement remains.
5	The server obtains the selected set $S_{t}$ from the Nash-stable action profile and sends local training instructions.
6	Each selected client minimizes (23) for *E* epochs and returns the compressed update $\Delta_{i}^{t}$.
7	The server computes weights $\alpha_{i}^{t}$ by (24) and updates $\theta_{t+1}=\theta_{t}+\sum_{i\in S_{t}}\alpha_{i}^{t}\Delta_{i}^{t}$.
8	The server updates cumulative participation $s_{i}^{t+1}$, fairness deficits $\rho_{i}^{t+1}$, and the reward budget for the next round.
Output	Final detector $\theta_{T}$ for IoT object detection.

**Table 3:** Overall detection performance over five independent runs.

Method	mAP@0.5	mAP@0.5:0.95	Cost	Fairness
Random-FL	45.6±0.5	27.8±0.4	1.00±0.02	0.71±0.02
FedAvg	47.9±0.5	29.4±0.4	0.96±0.02	0.73±0.02
FedProx	49.1±0.4	30.6±0.3	0.94±0.02	0.76±0.02
SCAFFOLD	49.7±0.4	31.0±0.3	0.98±0.03	0.78±0.02
FedNova	50.2±0.4	31.4±0.3	0.93±0.02	0.79±0.02
Oort-FL	50.5±0.4	31.7±0.3	0.88±0.02	0.77±0.02
Incentive-FL	50.8±0.3	31.9±0.3	0.86±0.02	0.82±0.02
Resource-FL	50.9±0.4	32.0±0.3	0.80±0.02	0.80±0.02
FedDet	51.1±0.3	32.3±0.3	0.92±0.02	0.77±0.02
GO-FedDet	52.0±0.3	33.2±0.2	0.74±0.01	0.88±0.01

*Note:* Bold values indicate the best result in each comparison.

**Table 4:** Impact of non-IID severity over five independent runs.

Label Skew	FedAvg	FedProx	GO-FedDet
0.1	50.4±0.3	51.0±0.3	52.8±0.2
0.3	48.6±0.4	49.7±0.3	52.0±0.3
0.5	46.2±0.5	47.8±0.4	50.4±0.3
0.7	43.5±0.5	45.1±0.4	48.1±0.4

*Note:* Bold values indicate the best result in each comparison.

**Table 5:** Communication and energy consumption.

Method	Upload (MB)	Energy (J)	Delay (s)
FedAvg	1280±21	912±18	184±5
FedProx	1280±20	925±17	189±5
SCAFFOLD	1410±24	960±21	201±6
FedNova	1265±19	904±17	178±5
Resource-FL	1043±18	792±15	154±4
GO-FedDet	946±15	731±13	139±4

*Note:* Bold values indicate the best result in each comparison.

**Table 6:** Sensitivity to key parameters.

Setting	mAP@0.5	Cost	Fairness
μ=0.001	50.7±0.4	0.73±0.02	0.86±0.02
μ=0.010	52.0±0.3	0.74±0.01	0.88±0.01
μ=0.100	50.9±0.4	0.75±0.02	0.87±0.02
$\lambda_{f}=0$	50.0±0.4	0.72±0.02	0.76±0.02
$\lambda_{f}=0.5$	51.4±0.3	0.73±0.02	0.84±0.02

*Note:* Bold values indicate the best result in each comparison.

## Data Availability

The original contributions presented in this study are included in the article. Further inquiries can be directed to the corresponding author.
